# Integrative path modeling and QTL mapping identify maturity, stem strength, and cell wall composition driving lettuce resistance to *Sclerotinia minor*

**DOI:** 10.1038/s41598-025-03775-1

**Published:** 2025-06-05

**Authors:** Ivan Simko, Bullo Erena Mamo, Shane L. Cantu, Hui Peng, Rebecca Grube Sideman, Ryan J. Hayes, Krishna V. Subbarao

**Affiliations:** 1 United States Department of Agriculture, Agricultural Research Service, Sam Farr United States Crop Improvement and Protection Research Center, Salinas, CA 93905 USA; 2 Department of Plant Pathology, University of California, Davis, Sam Farr United States Crop Improvement and Protection Research Center, Salinas, CA 93905 USA; 3 Department of Energy Great Lakes Bioenergy Research Center, Michigan State University, East Lansing, MI 48824 USA; 4 Everglades Research and Education Center – Horticultural Sciences Department, University of Florida, Belle Glade, FL 33430 USA; 5 Department of Agriculture, Nutrition and Food Systems, University of New Hampshire, Durham, NH 03824 USA; 6 Department of Agriculture, Agricultural Research Service, Forage Seed and Cereal Research Unit, United States, Corvallis, OR 97321 USA; 7 Department of Microbiology and Plant Pathology, University of California, Riverside, CA 92521 USA

**Keywords:** *Lactuca*, Lettuce drop, Xylose, Arabinose, Lignin, Callose, Stem strength, Plant breeding, Plant genetics, Biotic

## Abstract

Lettuce (*Lactuca sativa*) is highly vulnerable to *Sclerotinia minor*, the pathogen causing lettuce drop. Breeding for resistance is the most effective control strategy; however, full resistance has not been achieved, and current partial resistance sources are often linked with undesirable traits, such as early bolting. This study aimed to unravel the genetic basis of partial resistance to *S. minor* and its relationship with plant maturity (bolting), stem mechanical strength (SMS), and cell wall composition (CWC) using a recombinant inbred line (RIL) population derived from a cross between the susceptible iceberg cv. ‘Salinas’ and the resistant oil-seed accession PI 251246. Field evaluations indicated that resistance was linked to earlier bolting, stronger stems, and higher pentose content. Path analysis demonstrated that earlier-maturing plants exhibited increased resistance through enhanced SMS and modified CWC, particularly with higher xylose and lower arabinose levels. Further analysis indicated a significant relationship between syringyl lignin content and resistance, especially in plants with varying bolting responses. Three key quantitative trait loci (QTLs) on linkage groups (LG) 2, 6, and 7 were consistently associated with resistance, bolting, and SMS. Importantly, residual QTL analysis revealed that the resistance locus on LG7 acted independently of maturity, suggesting a distinct resistance mechanism. Callose synthase emerged as a key candidate gene within the LG7 resistance QTL, located near - but distinct from - genes associated with plant maturity and flowering. These findings provide valuable insights into decoupling resistance from early bolting, suggesting a pathway for breeding lettuce cultivars with improved disease resistance and delayed bolting.

**Supplementary Information:**

The online version contains supplementary material available at 10.1038/s41598-025-03775-1.

## Introduction

Lettuce (*Lactuca sativa*) is the world’s leading leafy vegetable but is highly susceptible to various diseases, including lettuce drop caused by the fungal pathogens *Sclerotinia minor* (and *S*. *sclerotiorum* to a lesser extent). Annual losses caused by lettuce drop have varied from < 1% to nearly 75%^1^. Factors such as the cropping history, irrigation and tillage methods, and other environmental and cultural characteristics of the field influence losses^2^. *Sclerotinia* spp. have evolved mechanisms such as the production of phytotoxins and cell wall-degrading enzymes (CWDEs), to overcome host immunity and extract nutrients from plant tissues^3–5^. These CWDEs particularly target host structures including the stem, facilitating rapid infection^6^.

In lettuce, soft basal stems have been identified as a susceptibility factor for *S. minor*^7^. Once infected, lettuce plants collapse rapidly, often within a few days^2,8^. Although cultural, antifungal, and biological control methods have been attempted^9,10^, managing lettuce drop remains challenging due to the pathogens’ ability to overwinter in soil as sclerotia and the windborne nature of the inoculum^1,11^. Host resistance is the most effective strategy for managing lettuce drop diseases, but it is currently limited. Complete resistance to *S. minor* in lettuce has not yet been identified, although certain accessions exhibit partial resistance.

A promising source of partial resistance to *S. minor* is the oilseed-type lettuce accession PI 251246, identified as one of the most resistant accessions among approximately 500 tested^7,12,13^. PI 251246 bolts earlier than almost all tested accessions, with bolting - defined as stem elongation - marking the transition from the vegetative to the reproductive stage in the plant’s life cycle. Upon bolting, PI 251246 produces stems with high mechanical strength^7^ associated with hemicellulose constituents and lignin polymers^14^, which are recalcitrant to the pathogen’s lignocellulolytic enzymes. However, the close relationship between PI 251246’s early bolting habit and resistance complicates the separation of these traits, raising the question of whether the resistance is due to a disease avoidance mechanism rather than physiological resistance^12,15^. In this context, avoidance is characterized as a mechanism that relies primarily on developmental or morphological traits that minimize contact between vulnerable plant tissues and potential sources of infection, such as early bolting.

Plants that reach the bolting stage earlier develop stronger, lignified stems that are less prone to degradation by fungal enzymes compared to the softer tissues of late bolting plants still in the rosette stage. Consequently, avoidance due solely to very early bolting, while reducing the likelihood of successful pathogen establishment, does not necessarily increase the plant’s physiological resistance. Furthermore, this mechanism may not be useful for developing new lettuce cultivars, as early bolting is generally an undesirable trait in commercial lettuce production. This complication underscores the need to distinguish between true physiological resistance and morphology-based avoidance mechanisms when breeding for disease resistance in lettuce. In research investigating this concern, bolted plants of PI 251246 inoculated with ascospores from *S. sclerotium* appeared to resist infection, suggested that they had a resistance mechanism separate or in addition to avoidance^16,17^.

To better understand the genetic basis of resistance in this accession, a biparental mapping population was developed from a cross between PI 251246 and the susceptible iceberg cv. ‘Salinas’^15^. This mapping population segregates for both resistance to *S. minor* and earliness of bolting. However, environmental factors, such as day length and temperature, further complicate the study, as bolting behavior in this population varies under different conditions^18^. To address this, resistance and bolting were evaluated across three growing seasons (spring, summer, and fall), and the summer experiment was also used to examine the relationship between bolting, cell wall composition (CWC), stem mechanical strength (SMS), and resistance. The objectives of this study were to analyze the interplay between these traits, identify quantitative trait loci (QTLs) associated with resistance, and determine a possible basis of resistance to *S. minor* independent of early bolting.

## Materials and methods

### Plant material

An F_7_ generation of recombinant inbred lines (RILs) of lettuce was used in this study. The RILs were developed from a cross between the iceberg cv. ‘Salinas,’ susceptible to lettuce drop, and the oilseed-type accession PI 251246, known for its high partial resistance to the disease^7,12,13^. After bolting, cv. ‘Salinas’ exhibits green stem coloration, while PI 251246 displays red to purple stem hues. The plants were grown in four field experiments, each artificially inoculated with *S. minor* sclerotia. Two of these experiments were conducted in the spring (SP02) and fall (FA02) of 2002, while the other two were conducted in summer 2017 (SU17). Inoculum production and field inoculation followed the procedures previously described^19^.

The experiments took place at the USDA-ARS experimental farm in Salinas, California, USA. The plants were grown on raised beds approximately 1 m wide and 25 centimeters high, following standard agricultural practices for the region. Each bed contained two parallel seedlines, spaced about 28 centimeters apart, with approximately 30 centimeters between plants within each seedline. The plants were monitored weekly throughout the growing season for the appearance of lettuce drop symptoms.

### Traits evaluation

In the 2002 experiments, a randomized complete block design (RCBD) was employed with four replications, and each plot contained approximately 25–30 plants. Lettuce seeds of 87 RILs were first sown in plug trays in a greenhouse, and four-week-old seedlings were transplanted into the *S. minor*-inoculated field. The RILs were assessed for the percentage of plants displaying symptoms of the disease 28 days after transplanting. Bolting, which indicates plant maturity, was evaluated on a scale from 0 to 5 using the plants remaining at the end of the experiment. The rate of bolting was assessed using a scale where 0 = no bolting, 1 = beginning to bolt, 2 = advanced bolting, 3 = beginning to flower, 4 = full flowering, and 5 = seed set.

In 2017, two additional experiments were conducted, each utilizing an RCBD with three replications and 30–35 plants per plot. One experiment involved all 159 RILs plus parental lines, while the other focused on a randomly selected subset of 30 RILs, which were used to calculate the broad-sense heritability (*H²*) of traits evaluated in both 2017 experiments. The larger experiment (SU17) was assessed for disease incidence and bolting rate. Additionally, resistance to lettuce drop was evaluated using disease severity index (DSI)^7^, and bolting was assessed by calculating the standardized area under the bolting progress stairs (sAUBPS)^20^. DSI data were derived from disease incidence and disease severity scores on a scale of 1 (indicating no degradation) to 5 (indicating complete degradation) of the basal stems of infected or dead plants. sAUBPS was calculated from weekly evaluations of bolting on the 0 to 5 scale. These improved methods accounted for the progression of bolting rather than just the final percentage of bolted plants and combined disease incidence and symptom severity by evaluating stem-level degradation rather than solely the percentage of symptomatic plants. Thus, sAUBPS and DSI were used for further statistical analyses in the SU17 experiment. Moreover, data on bolting and resistance from the three experiments were combined into a single trait for each characteristic. In the first step, data from individual experiments were transformed using the inverse normal transformation (INT) approach. Subsequently, the mean INT values for bolting and resistance were calculated by averaging data from the three experiments. These averaged data were then used alongside the original data from individual experiments in subsequent statistical analyses and QTL mapping.

When cv. ‘Salinas’ reached harvest maturity, stems from the RILs were collected and used to evaluate stem mechanical strength (SMS), stem cell wall composition (CWC), and stem color (categorized as green = 1, segregating = 2, or red/purple = 3). The presence of red/purple stem color indicates the accumulation of anthocyanins in the plant tissue^13^. The smaller experiment was used to evaluate a subset of traits: sAUBPS, DSI, and SMS parameters.

### Stem mechanical strength

Three parameters were used to assess SMS: cortex strength, xylem strength, and pith strength. These analyses were conducted using the TA.XTplus Texture Analyzer (Texture Technologies Corp., Hamilton, Massachusetts, USA/Stable Micro Systems, Godalming, Surrey, U.K.), following the manufacturer’s instructions. A detailed description of the procedures, including step-by-step instructions, was previously published^7^.

### Stem enzymatic digestion

Stem sampling for CWC involved cutting each plant at the stem base, with 8.5 centimeters of the stem above the base being harvested. This sampling length was chosen because it represents the region of the stem most in contact with the pathogen and, thus, the primary site of infection^7,14^. Samples were frozen in liquid nitrogen, lyophilized, and stored at -20 °C until further processing. Lyophilized stem samples were ground using a Thomas Wiley Mini-Mill with a 40-mesh screen (Thomas Scientific, Swedesboro, New Jersey, USA) and then submitted to two laboratories for analysis. The Great Lakes Bioenergy Research Center at Michigan State University, East Lansing, Michigan, USA performed digestibility tests^21^, which involved enzyme digestion and sugar analysis, yielding levels of glucose and pentose released after digestion with Accellerase 1000 (Genencor, Rochester, New York, USA). In this assay, glucose primarily comes from the breakdown of cellulose while pentose is a collective term referring to mostly xylose and arabinose released from the breakdown of hemicellulose in cell walls. These assays were originally developed to study potential biofuel production but were adapted in this study to mimic the digestion of plant tissues by the pathogen’s degradative enzymes.

### Stem glycosyl composition

The Complex Carbohydrate Research Center at the University of Georgia, Athens, Georgia, USA conducted glycosyl (arabinose, ribose, rhamnose, fucose, xylose, glucuronic acid, galacturonic acid, mannose, galactose, and glucose) and lignin composition analyses on samples from 128 RILs (with 31 samples omitted due to cost constraints). Glycosyl composition analysis was performed using gas chromatography/mass spectroscopy (GC/MS) of the per-O-trimethylsilyl (TMS) derivatives of the monosaccharide methyl glycosides. Myo-inositol (20 µg) was used as an internal standard for quantification. The TMS derivatives were produced from the samples by acidic methanolysis, followed by acetylation and TMS treatment^22^. GC/MS analysis was performed using an Agilent 7890A GC interfaced with a 5975C MSD (Agilent Technologies, Santa Clara, California, USA), utilizing a Supelco Equity-1 fused silica capillary column (30 m × 0.25 mm) (Supelco, Bellefonte, Pennsylvania, USA). Results were expressed as molar percentages (Mol %), calculated by determining the mass using the response factor and dividing by the original sample mass. Total carbohydrate content was expressed as a percentage by weight. Results for ribose, fucose, and glucuronic acid were not considered for statistical analyses and QTL mapping as almost all samples yielded values below the threshold detection levels.

### Stem lignin composition

For lignin analysis, samples were prepared in duplicate by weighing approximately 1.0 to 3.0 mg into stainless steel cups, which were then single-shot pyrolyzed (Frontier Lab, Sacramento, California, USA) at 500 °C to produce volatile compounds. These volatile compounds were analyzed for lignin content using a molecular beam mass spectrometer (Extrel Core Mass Spectrometers, Pittsburgh, Pennsylvania, USA). The raw data were processed using UnscramblerX 10.1 software (CAMO Software, Oslo, Norway) to obtain the principal components and raw lignin data. Additionally, NIST 8492 (lignin content, 26.2%) and Aspen standards were pyrolyzed and analyzed in the same batch as the unknown samples. Both standards were used for data quality control, with NIST 8492 also being employed to correct the raw lignin data. The composition was determined for the content of syringyl, guaiacyl, p-hydroxylphenyl (henceforth referred to as hydroxyphenyl), and the total lignin content.

### Statistical analyses

Chi-square tests were performed to assess the segregation of stem color (green or red/purple). Pearson’s linear correlation coefficients were calculated to assess the relationships among all pairs of evaluated traits. P-value adjustment for multiple comparisons was performed using the false discovery rate (FDR) method. Principal component analysis (PCA) was conducted to further assess the relationships among the traits. To identify the most influential traits associated with lettuce drop resistance in the SU17 experiment, partial least squares (PLS) regression was employed. This approach was particularly useful for handling multicollinearity among traits, reducing dimensionality, and focusing on variables with variable importance in projection (VIP) scores greater than 1. Random forest (RF) analysis was conducted to rank the importance of traits in predicting lettuce drop resistance, with traits contributing more than 1% retained for further analysis. RF analysis was employed as a complementary method to identify variables associated with resistance because of its ability to handle high-dimensional datasets and nonlinear relationships. Moreover, RF is resistant to overfitting due to its bootstrapping and aggregation process, which makes it robust across different datasets, providing stable and reliable rankings of important traits.

Results from PLS (VIP > 1) and RF (contribution > 1%) analyses were considered when developing the path analysis model, focusing on traits that contributed the most to resistance while reducing noise from less relevant variables. Path analysis was then used to model the direct and indirect effects of the selected traits on lettuce drop resistance. The path model was refined by including all significant traits and gradually eliminating non-significant relationships. The model with the lowest Akaike information criterion (AIC) and root mean square error of approximation (RMSEA) was selected for interpretation. Statistical analyses were conducted using JMP Pro v.17 (SAS Institute, Cary, North Carolina, USA). Broad-sense heritability (*H*^*2*^) was calculated from the two 2017 experiments using variance components estimated by the SAS “proc varcomp” procedure as *H*^*2*^ = *σ*^*2*^_G_ /[*σ*^*2*^_G_ + (*σ*^*2*^_E_ /*e*) + (*σ*^*2*^_e_ /*re*)] where *σ*^*2*^_G_ is the variance component for genotype, *σ*^*2*^_E_ is the variance component for experiment, *σ*^*2*^_e_ is the residual variance, *r* is the number of replications in each experiment, and *e* is the number of experiments.

### Genotyping and linkage map construction

Genotyping and linkage map construction of the mapping population were performed as described previously^18^. DNA was extracted from each RIL using the DNeasy Plant Mini Kit (Qiagen, Valencia, California, USA), digested with EcoT22I, and processed through genotyping-by-sequencing (GBS)^23^ at the Cornell Institute of Biotechnology (http://www.biotech.cornell.edu/brc/genomics-facility). Single nucleotide polymorphism (SNP) identification and filtering were conducted using the TASSEL pipeline^24^. The linkage map was generated with MSTmap (http://mstmap.org/), where fully linked markers were collapsed into single combined markers. Four additional SNP-based ‘LZ’ markers, previously developed for mapping loci related to bolting and flowering earliness in lettuce, were also included^18^. In total, 757 molecular markers spanning nine linkage groups (LGs) corresponding to the nine lettuce chromosomes were used for QTL mapping.

### QTL analysis

QTL analyses were performed on both the original data and residuals from the regression to maturity (bolting). Various models (e.g., polynomial, sigmoid, and exponential) were tested for curve fitting, with the best fit determined using AIC. Residuals were used to detect QTLs for the analyzed traits independent of plant maturity (bolting). The analyses were conducted using QGene v. 4.4.0 software^25^ with the multiple interval mapping (MIM) approach and a 1 cM scan interval. The genome-wide significance threshold for QTL detection was set at α = 0.05, determined through 1,000 permutations.

### Candidate genes identification

Candidate genes within the 1-LOD support intervals of significant QTLs were identified using the latest version of the lettuce reference genome assembly (Lsat_Salinas_v11) available at NCBI (https://www.ncbi.nlm.nih.gov/datasets/genome/GCA_002870075.4/). The physical range of each 1-LOD support interval was determined by aligning the sequences of QTL flanking markers to the reference genome. Gene annotation information was obtained from published data^26^ and is available online in the NCBI reference genome.

## Results

### Evaluations of bolting and resistance

The three field experiments were conducted at different times of the year, resulting in variations in average daylength, which influenced plant growth and bolting earliness. The average daylength was approximately 14.5 h in SP02, 13 h in SU17, and 11.5 h in FA02. Correspondingly, the average bolting scores were 1.56 ± 0.14 in SP02, 1.54 ± 0.07 in SU17, and 0.83 ± 0.13 in FA02. The disease incidence scores also differed slightly across experiments, with average values of 27.9 ± 1.5 in SP02, 24.3 ± 0.8 in SU17, and 24.4 ± 0.8 in FA02. In SU17, the average values of sAUBPS and DSI were 1.53 ± 0.04 and 66.5 ± 1.2, respectively. Broad-sense heritability (*H²*), estimated from two SU17 experiments, showed high heritability for most traits: 0.97 for sAUBPS (bolting), 0.87 for DSI (disease severity), 0.85 for xylem strength, and 0.75 for pith strength. The only exception was cortex strength, with an *H²* of 0.43.

### Relationships between traits

**Fig. 1:**
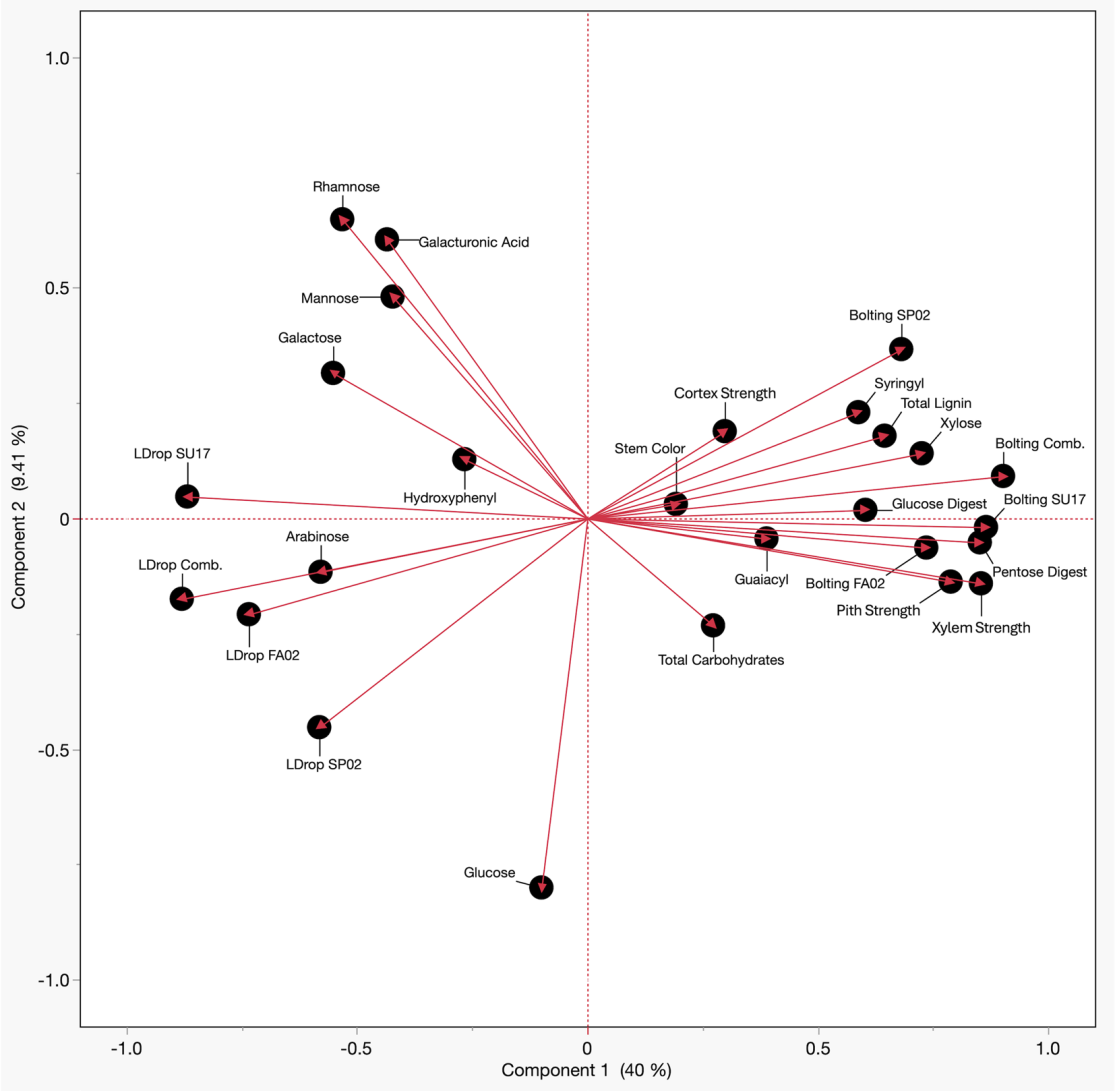
Principal component analysis (PCA) of variables evaluated in the 2002 (SP02, FA02) and 2017 (SU17) experiments. The arrows indicate the direction and strength of each variable’s contribution to the principal components, with longer arrows representing stronger contributions. Arrows pointing in the same direction for variables indicate a positive correlation, suggesting that as one variable increases, the other also tends to increase. Conversely, arrows pointing in opposite directions indicate a negative correlation, meaning that as one variable increases, the other tends to decrease. The grouping of data points shows the clustering of variables.

All traits, except glucose content in the stem cell wall, showed significant correlations (*p* < 0.05) with lettuce drop severity (Table 1). Bolting had the strongest negative correlation (*r* = -0.816, *p* = 5.55e-38), followed by xylem strength (*r* = -0.755, *p* = 1.18e-29) and pentose content (*r* = -0.708, *p* = 1.20e-24). PCA confirmed these relationships, grouping lettuce drop severity data across experiments (LDrop) and showing a strong negative correlation with bolting, xylem strength, pith strength, and pentose content, while arabinose was positively correlated (Fig. 1). PLS regression in the SU17 experiment identified seven traits as significantly related to lettuce drop severity (VIP > 1). Bolting had the highest VIP score (VIP = 1.717), followed by xylem strength (VIP = 1.526), pith strength (VIP = 1.422), and pentose content (VIP = 1.348). RF analysis further supported these findings, indicating that bolting contributed 64.0% to the prediction of resistance, with xylem strength contributing 15.1%, and pentose content 4.2%. Other traits contributed less than 3% individually (Table 1).

**Table 1:** Relationship between traits and lettuce drop disease severity index (DSI) in 2017 (SU17) in the ‘Salinas’ × PI 251246 recombinant inbred line population.

Trait	*N*	Linear correlation (*r*)	Linear correlation (*p*-value)^1^	PLS (VIP)^2^	Random Forest (contribution %)^3^
Bolting SU17	159	-0.816	5.55e-38	1.717	64.0
Cortex strength	159	-0.204	1.26e-2	0.485	1.0
Xylem strength	159	-0.755	1.18e-29	1.526	15.1
Pith strength	159	-0.688	5.52e-23	1.422	2.3
Stem color	159	-0.186	2.12e-2	0.652	0.6
Glucose (digest)	159	-0.492	1.50e-10	0.912	2.0
Pentose (digest)	159	-0.708	1.20e-24	1.348	4.2
Arabinose	128	0.504	3.48e-9	1.017	1.4
Rhamnose	128	0.445	2.75e-7	0.936	0.6
Xylose	128	-0.620	2.40e-14	1.181	1.9
Galacturonic acid	128	0.389	8.81e-6	0.786	1.0
Mannose	127	0.358	5.25e-5	0.732	0.5
Galactose	128	0.479	2.61e-8	0.926	0.6
Glucose	128	0.034	7.05e-1	0.116	0.4
Total carbohydrates	128	-0.178	4.69e-2	0.597	1.0
Total lignin	128	-0.454	1.60e-7	1.027	1.1
Syringyl	128	-0.405	3.59e-6	0.940	0.6
Guaiacyl	128	-0.287	1.41e-3	0.702	0.8
Hydroxyphenyl	128	0.219	1.57e-2	0.525	0.9

^1^P-values were adjusted for multiple comparisons using the false discovery rate (FDR) approach.

^2^Partial least square (PSL) variable importance projection (VIP) values over 1 were considered important.

^3^Random forest (RF) analysis was performed using 10,000 trees.

### Path analysis

**Fig. 2:**
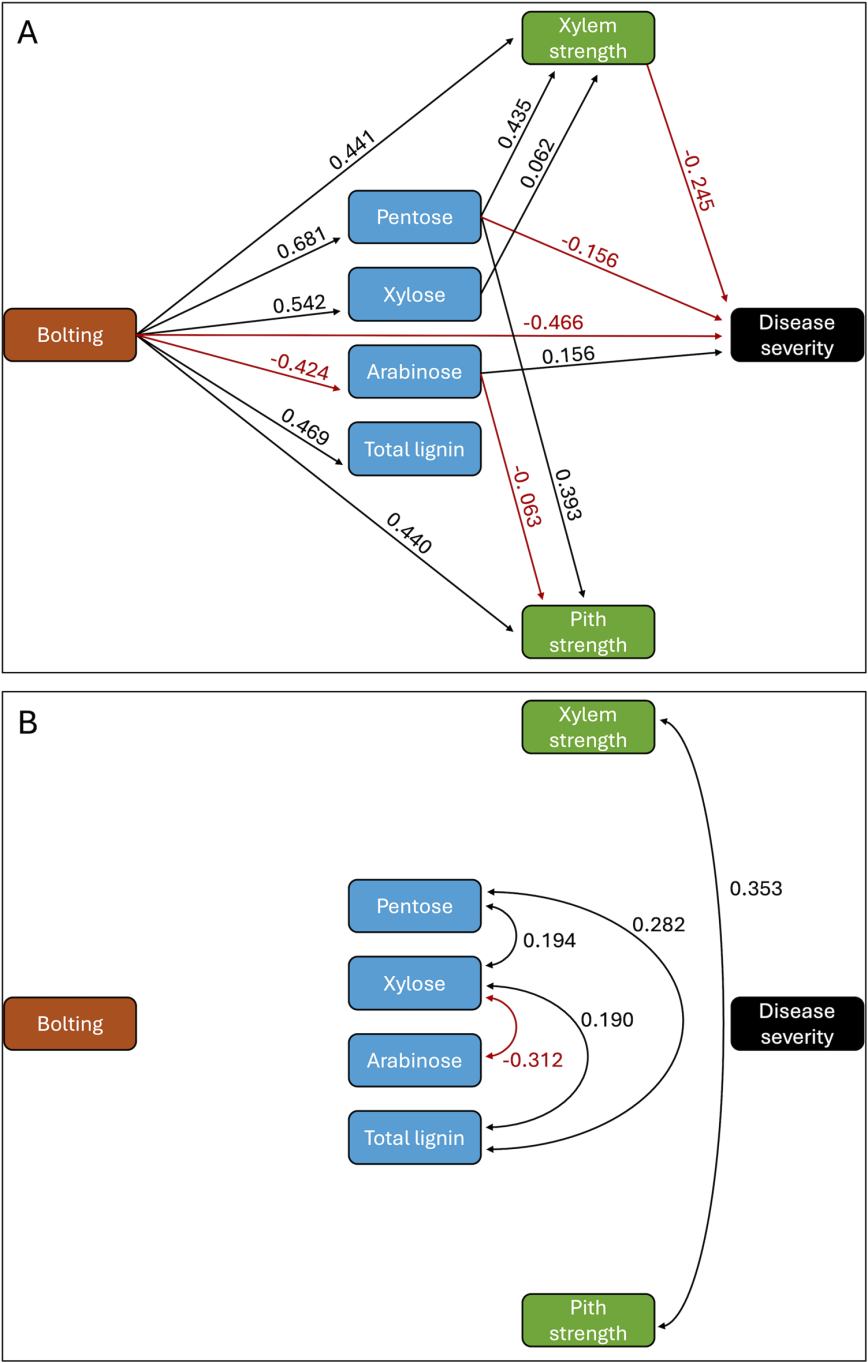
Path analysis model illustrating the effect of bolting, stem cell wall composition, and stem strength on lettuce drop severity, evaluated in 2017 (SU17). The final path model was selected based on optimal fit criteria, specifically the lowest Akaike information criterion (AIC) and root mean square error of approximation (RMSEA) values. The model was divided into two parts due to its complexity: **Part A (top panel)**: Standardized regression values show the strength and direction of relationships or paths between predictor (independent) and outcome (dependent) variables. Higher values indicate stronger effects, with positive values reflecting direct relationships and negative values indicating inverse relationships. **Part B (bottom panel)**: Covariances between pairs of variables quantify the extent to which two variables vary together. Positive covariances suggest that the two variables tend to increase together, while negative covariances imply an inverse relationship (e.g., as xylose increases, arabinose tends to decrease).

Based on the results of PLS and RF analyses, key traits from the SU17 experiment were included in the final path model: bolting score (maturity), pentose and glucose content from digestibility assay, arabinose, xylose, and total lignin content, and xylem and pith strength. Path analysis revealed that bolting directly influenced all other traits. Plants that bolted earlier exhibited higher pentose content, lower arabinose levels, greater xylem strength, and increased resistance to lettuce drop (Fig. 2a). Additionally, xylem strength had a direct positive effect on resistance, while pith strength influenced resistance indirectly through xylem strength. Xylose and total lignin content also had indirect effects, largely through their associations with pentose and arabinose (Fig. 2b), with xylose also contributing directly to stronger xylem. Since the digestibility assay measures pentose, primarily from xylose in hemicellulose, alternative path models tested the inclusion of either pentose or xylose individually. Nevertheless, the model containing both pentose and xylose provided the best fit. Though the largest standardized residuals between predicted and observed correlations were observed for arabinose-pentose content (-1.24), disease severity-xylose content (-0.86), and pith strength-total lignin (0.76) (Supplementary Fig. 1), these values are considered relatively small, indicating a good overall model fit. It is important to note that, while path analysis provides a statistical perspective, it does not necessarily represent a biological model; however, it does offer insights into trait interactions.

### QTL analysis

QTL analysis was conducted for the traits of bolting, resistance, stem color, SMS, and CWC attributes. Residual QTLs, derived from regressions related to bolting, were also examined to identify QTL locations independent of maturity. The best-fitting curves for these traits were frequently modeled using the four-parameter probit function; thus, this function was employed to calculate residuals for all traits.

**Fig. 3:**
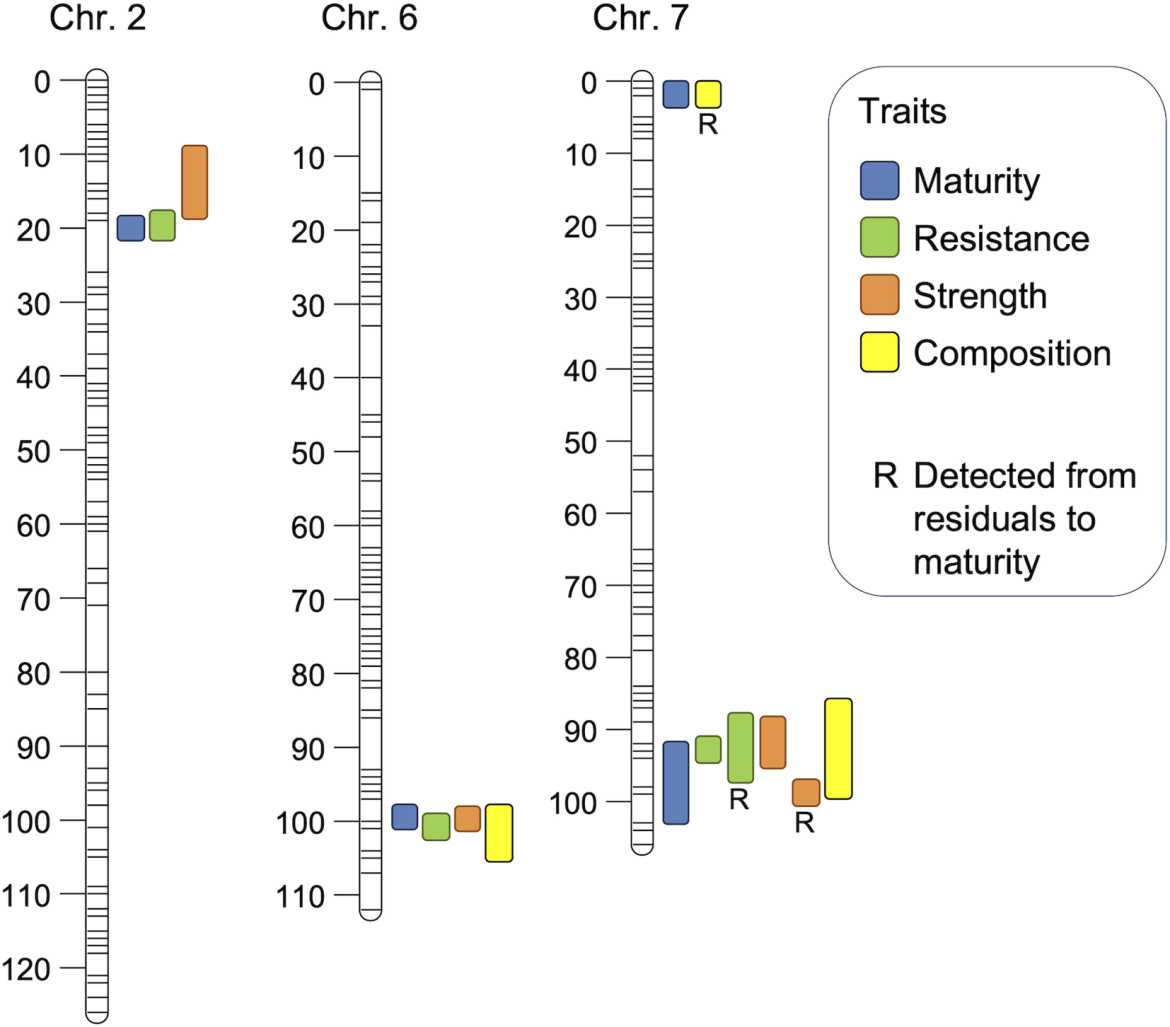
Colocation of QTLs associated with maturity, resistance to lettuce drop, stem strength, and stem cell wall composition in the ‘Salinas’ × PI 251246 RIL population. Each bar represents the QTL range for all traits and/or experiments within a specific trait category, including the 1-LOD support interval. For visual clarity, the minimum bar length is set to approximately 4 cM, even if the actual support interval is smaller. The letter ‘R’ indicates QTLs for traits derived from residuals relative to maturity. Exact QTL locations for individual traits and experiments are provided in Table 2.

Three major QTLs associated with maturity (bolting) were detected on LG2 (20.2–21.0 cM), LG6 (99.7–100.7 cM), and LG7 (90.2–103.6 cM), with a minor QTL on LG7 (1.1–2.0 cM) detected in FA02 (Table 2). The same major QTL regions were also linked with lettuce drop resistance on LG2 (17.7–21.0 cM), LG6 (100.0–101.9 cM), and LG7 (90.8–95.2 cM). Similar genomic locations were associated with xylem and pith strengths on LG2, LG6, and LG7; whereas, the same LG2 region was detected for cortex strength (Fig. 3). QTLs for stem cell wall components (xylose, arabinose, mannose, pentose and glucose from digestion assay, total lignin, or syringyl) were also found at overlapping locations on LG6 (97.4–104.9 cM) and LG7 (84.8–99.5 cM), reinforcing the relationships between plant maturity, CWC, SMS, and lettuce drop resistance identified by correlation, PCA, PLS, RF, and path analyses.

**Table 2:** QTLs for lettuce drop resistance, rate of bolting, stem mechanical strength, cell wall composition, and anthocyanin content in the ‘Salinas’ × PI 251246 recombinant inbred line population.

Category	Trait^1^	Nearest marker^2^	LG	Location (cM)	Support interval (cM)^3^	LOD	Variance (*R*^2^)^4^	Allele^5^
Maturity	Bolting SU17	LZ001	2	21.0	20.2–21.0	12.6	30.4	Sal
Maturity	Bolting comb.	LZ001	2	21.0	21.0–21.0	9.5	23.8	Sal
Resistance	LDrop FA02	cS2_42117828	2	17.7	17.7–18.0	4.4	11.9	PI
Resistance	LDrop SU17	cS2_40045553	2	18.1	17.7–21.0	7.4	19.2	PI
Resistance	LDrop comb.	cS2_42117828	2	17.7	17.7–17.9	7.8	20.1	PI
Strength	Cortex strength	cS2_40045553	2	18.1	17.7–18.2	3.7	10.2	Sal
Strength	Xylem strength	cS2_28785120	2	10.3	9.9–10.5	7.1	18.4	Sal
Strength	Pith strength	cS2_23073172	2	8.1	7.9–8.3	3.9	10.6	Sal
Anthocyanin	Stem color – resid.	S5_401233533	5	170.8	170.3–170.8	7.0	15.7	PI
Maturity	Bolting SU17	LZ175	6	99.7	99.7–100.7	11.1	27.4	PI
Maturity	Bolting comb.	LZ175	6	99.7	99.7–100.7	13.0	31.2	PI
Resistance	LDrop SU17	cS6_220422004	6	100.3	100.0–101.9	11.1	26.8	Sal
Resistance	LDrop comb.	cS6_220422004	6	100.3	100.3–100.3	9.8	24.6	Sal
Strength	Xylem strength	S6_221717112	6	100.7	99.7–101.0	6.2	16.3	PI
Strength	Pith strength	S6_221717112	6	100.7	99.7–101.0	4.6	12.5	PI
Composition	Glucose (digest)	S6_227148077	6	104.4	101.8–104.9	3.6	9.5	PI
Composition	Pentose (digest)	cS6_220422004	6	100.3	99.7–102.3	7.3	19.0	PI
Composition	Arabinose	LZ175	6	99.4	97.4–99.4	3.9	10.5	Sal
Composition	Xylose	cS6_220422004	6	100.3	100.0–100.4	6.8	17.8	PI
Composition	Total lignin	LZ175	6	99.4	97.6–99.4	4.8	12.9	PI
Composition	Syringyl	LZ175	6	99.4	97.6–100.4	4.5	12.1	PI
Maturity	Bolting FA02	S7_3112321	7	1.5	1.1–2.0	3.1	8.5	PI
Composition	Total lignin – resid.	cS7_1980750	7	0.0	0.0–1.1	3.4	9.3	Sal
Composition	Syringyl – resid.	cS7_1980750	7	0.0	0.0–1.1	3.7	10.0	Sal
Maturity	Bolting SP02	cS7_232556350	7	103.5	102.8–103.6	6.1	16.0	PI
Maturity	Bolting SU17	cS7_209035609	7	94.1	94.1–95.0	10.8	26.6	PI
Maturity	Bolting comb.	cS7_204555011	7	93.2	92.0–93.6	13.7	32.6	PI
Resistance	LDrop SP02	cS7_209035609	7	94.1	93.7–94.2	10.3	25.5	Sal
Resistance	LDrop FA02	cS7_209035609	7	94.1	93.6–95.2	6.7	17.6	Sal
Resistance	LDrop SU17	cS7_200076206	7	91.9	90.8–91.9	9.6	24.1	Sal
Resistance	LDrop comb.	cS7_200076206	7	91.9	91.3–92.0	17.4	39.1	Sal
Resistance	LDrop SP02 – resid.	cS7_191583577	7	86.7	86.6–86.8	4.8	13.0	Sal
Resistance	LDrop FA02 – resid.	cS7_209035609	7	94.1	93.7–97.4	4.7	12.6	Sal
Resistance	LDrop comb. – resid.	cS7_191583577	7	86.7	86.6–86.8	5.4	14.4	Sal
Strength	Xylem strength	cS7_209035609	7	94.1	93.5–95.6	8.5	21.7	PI
Strength	Pith strength	S7_195299849	7	88.6	88.2–90.8	7.2	18.6	PI
Strength	Xylem strength – resid.	cS7_223434304	7	98.3	98.2–98.5	5.4	14.4	PI
Strength	Pith strength – resid.	cS7_223434304	7	98.3	98.2–98.5	4.1	11.3	PI
Composition	Pentose digest	S7_191007094	7	86.3	84.8–86.4	5.5	14.6	PI
Composition	Xylose	cS7_209035609	7	94.1	93.5–95.6	5.1	13.7	PI
Composition	Mannose	cS7_224771436	7	98.7	98.6–99.5	8.6	21.9	Sal
Composition	Total lignin	LZ093	7	86.5	85.8–88.2	3.1	8.6	PI
Anthocyanin	Stem color	cS9_169472460	9	91.9	90.1–91.9	9.6	24.2	PI
Anthocyanin	Stem color – resid.	cS9_169472460	9	91.9	90.8–91.9	17.0	38.7	PI

^1^QTLs identified from residuals of the traits calculated from the regression to bolting are indicated as ‘resid.’

^2^Molecular marker nearest to the QTL.

^3^The range of 1-LOD support interval.

^4^Percent of the total phenotypic variation of the trait explained by the QTL.

^5^Parent contributing alleles that increase the trait value (Sal – cv. ‘Salinas’, PI – accessions PI 251246). Higher trait values mean earlier bolting, higher disease incidence or severity rating, stronger stems, and higher content of evaluated compounds.

QTLs calculated from residuals to maturity did not identify any QTLs on LG2 or LG6, but QTLs for resistance and stem strength were detected on LG7 (86.6–98.5 cM). The analysis using residual CWC identified a single QTL for each total lignin and syringyl on LG7 (0.0–1.1 cM), matching the location of a minor maturity QTL.

In each QTL where maturity, resistance, and stem strength were mapped to the same chromosomal region, alleles for earlier bolting were associated with lower disease ratings and stronger stems (Table 2). These characteristics were linked with alleles for higher levels of pentose and glucose from digestion assays, and higher contents of xylose, total lignin, and syringyl, but lower levels of arabinose and mannose. In three QTLs on LG6 and LG7, alleles associated with earlier bolting (maturity), higher resistance (lower disease rating), stronger stems, and the associated cell wall constituents originated from PI 251246: whereas, in the QTL on LG2, these traits were associated with alleles from cv. ‘Salinas’.

For stem color, which indicates anthocyanin content, a QTL on LG9 (90.1–91.9 cM) was identified. Interestingly, when residuals of color (from regression related to bolting) were analyzed, two QTLs were detected: one on LG9 at the same location as the original (90.8–91.9 cM) but with a higher LOD score, and another at the proximal end of LG5 (170.3–170.8 cM) (Table 2).

### Integration of findings

The integration of QTL and path analysis results highlights important genomic regions on LG2, LG6, and LG7 that contribute to maturity, resistance, SMS, and CWC traits. The colocation of QTLs for these traits suggests that genes with pleiotropic effects may reside in these regions, simultaneously influencing multiple traits. In particular, LG6 and LG7 appear to contain key loci controlling bolting, SMS, CWC, and resistance.

The study identified residual QTLs on LG7 that enhance resistance to lettuce drop and SMS, independently of the maturity factor. Path analysis indicates a correlation between earlier bolting and increased pentose levels, as well as stronger stems and greater resistance to lettuce drop. The presence of significant QTLs for both stem mechanical strength and resistance suggests that these traits are directly affected by genetic factors outside the scope of maturity.

### Candidate genes

A search of the genomic regions surrounding four significant QTLs identified several characterized genes with presumed functions as well as uncharacterized loci. The major QTL region on LG2 contained 108 genes, LG6 contained 360 genes, and LG7 contained 396 genes, while the minor QTL on the distal end of LG7 included 8 genes (Supplementary Table 1). Based on known or predicted functions, the QTL on LG2 contained 13 putative candidate genes potentially involved in plant bolting, resistance, stem cell wall composition, and stem strength. These included *Flowering Locus T*, *Auxin response factor 5*, *Disease resistance protein RPV1*, *RGA3*, *TMV resistance protein N*, and *Pathogenesis-related protein 1B*. The QTL on LG6 contained 32 candidate genes, such as *Abscisic acid 8’-hydroxylase 4*, *Ethylene-responsive transcription factor ERF012-like*, *RAP2-13*, *Extensin-like proteins*, *F-box/LRR-repeat protein 3*, *Sucrose-phosphate synthase 4*, and *Wall-associated receptor kinase 2*. The QTL on LG7 contained 18 genes, including *Callose synthase 5-like*, *Constans-like 9 (COL9)*, *Disease resistance protein RGA5*, *Ethylene-responsive transcription factor 4*, *Fasciclin-like arabinogalactan protein 2*, *Gibberellin 2-beta-dioxygenase 1*, *Pathogen-related protein*, and *Phytochrome C-like*. The minor QTL on LG7 included 4 genes, such as *E4 SUMO-protein ligase PIAL1* and *WRKY transcription factor 22* (Table 3). Several of these candidate genes may be involved in multiple traits, such as *Callose synthase*, which could affect cell wall composition, stem mechanical strength, and disease resistance.

**Table 3:** List of notable candidate genes potentially involved in plant bolting, resistance, stem strength, and cell wall composition detected at four significant QTL locations.

QTL	Candidate gene^1^
LG2	Aluminum-activated malate transporter 9
	Auxin response factor 5 (ARF5)
	Cleavage stimulating factor 64
	Disease resistance protein RPV1
	Flowering locus T – like FT
	Kinesin-like protein KIN-6
	Pathogenesis-related protein 1B
	Protein HEADING DATE 3 A (*LsFT*)^2^
	Protein translocase subunit SECA2
	Putative disease resistance protein RGA3
	Secreted RxLR effector protein 161-like
	TMV resistance protein N
	Tubulin beta chain/tubulin beta-6 chain-like
	Two-component response regulator ARR14
LG6	1-aminocyclopropane-1-carboxylate oxidase
	Abscisic acid 8’-hydroxylase 4
	Agamous-like MADS-box protein AGL11, AGL24, AGL104
	Alpha-amylase
	Annexin Gh1
	Beta-ketoacyl-[acyl-carrier-protein] synthase III A,
	BRI1 kinase inhibitor 1
	Classical arabinogalactan protein 5
	Cryptochrome-1
	Cyclin-D4-1, cyclin-D5-3
	Cytochrome P450 81Q32, 98A2
	E3 ubiquitin-protein ligase HOS1-like, PRT6-like
	Endo-1,4-beta-xylanase 1
	Ethylene-responsive transcription factor ERF012-like, RAP2-13
	Extensin-like proteins
	F-box/LRR-repeat protein 3
	Glutamate receptor 2.9
	Immune-associated nucleotide-binding protein 9
	MADS-box transcription factor 23
	Major allergen Pru ar 1-like
	MFP1 attachment factor 1
	Patatin-like protein 1, 2
	Probable beta-1,4-xylosyltransferase IRX9H
	Probable pectin methyltransferase QUA2
	Protein DETOXIFICATION 27, 48
	RING-H2 finger protein ATL58
	Serine/threonine-protein kinase CTR1, SAPK2
	Sucrose-phosphate synthase 4
	UDP-galactose transporter 1
	UDP-glucuronate alpha-glucuronosyltransferase 2
	Vacuolar protein sorting-associated protein 52 A
	Wall-associated receptor kinase 2
LG7 (minor)	E4 SUMO-protein ligase PIAL1
	Exocyst complex component SEC10b
	Pentatricopeptide repeat-containing protein At3g61520
	WRKY transcription factor 22
LG7	9-cis-epoxycarotenoid dioxygenase NCED1
	B-box zinc finger protein 23
	BES1/BZR1 homolog protein 4
	Callose synthase 5-like
	Cold-responsive protein kinase 1
	Constans like-9 COL9 (*LsCOL9*)
	Disease resistance protein RGA5
	Ethylene-responsive transcription factor 4
	Exopolygalacturonase
	Fasciclin-like arabinogalactan protein 2
	Gibberellin 2-beta-dioxygenase 1
	Nudix hydrolase 2
	Pathogen-related protein
	Peroxisomal fatty acid beta-oxidation multifunctional protein AIM1
	Phytochrome C-like (*LsPhyC*)
	Polyphenol oxidase I
	Probable beta-1,3-galactosyltransferase 14
	Probable hexosyltransferase MUCI70

^1^A complete list of identified genes and their locations is included in Supplementary Table 1.

^2^Detected in a previous study^18^ within the 1.5-LOD support interval of QTLs found in the current study.

## Discussion

### Trait associations with resistance

Our study aimed to decouple early bolting from resistance to *S. minor* in lettuce by employing integrative path modeling and QTL analysis. We sought to identify the key traits associated with resistance within the ‘Salinas’ × PI 251246 population. The analysis revealed significant correlations between resistance to lettuce drop and several key traits, with earlier bolting, stronger stems, and higher pentose content playing crucial roles in enhancing resistance in the population. These findings align with previous research, which highlighted strong associations between resistance and traits including stem strength, higher xylose, and lignin content—especially syringyl lignin—and a negative correlation with arabinose content^14^. Through path analysis, we confirmed that bolting (stem elongation) has a direct and notable effect on resistance. The results show that early maturing plants are more disease resistant, likely due to the enhancement of stem mechanical strength (including both xylem and pith strength) and changes in stem cell walls composition, which feature higher levels of xylose, lignification, and lower levels of arabinose. These direct and indirect effects indicate that enhancing SMS and optimizing CWC could serve as valuable strategies for breeding lettuce with greater resistance to lettuce drop.

### Pentose and xylose correlation with resistance

Pentose content, measured via digestion assays, is expected to mainly represent xylose^21^, as the ratio of xylose to arabinose is high in lettuce^14^. While both high pentose and high xylose levels correlated significantly with increased resistance, pentose displayed a stronger correlation with DSI (*r* = -0.71) than xylose (*r* = -0.62), a difference also confirmed by path analysis. Notably, this discrepancy did not result from fewer RILs being analyzed for xylose content; recalculating pentose correlations for a smaller subset of samples produced the same result as the full dataset. The stronger correlation between pentose content and resistance suggests that pentose, as detected by digestibility assays, may better capture the overall integrity and complexity of the cell wall compared to the more targeted measurement of xylose. The enzyme-based digestion used in pentose assays could more closely mimic pathogen-induced cell wall breakdown, thus better representing the cell walls’ ability to resist degradation. Pentose sugars, including xylose, contribute to hemicellulose-lignin cross-linking, which affects cell wall structure^27^, and this cross-linking may explain why pentose content correlates more strongly with resistance than xylose alone.

### Resistance QTLs identification

The identification of QTLs on linkage groups LG2, LG6, and LG7 for traits such as bolting, SMS, and CWC suggests a genetic basis for these correlations with resistance. The colocation of these QTLs with those for lettuce drop resistance supports the idea that bolting time, stem mechanical strength, and cell wall composition are genetically linked to disease resistance. The major resistance QTL on LG7, identified in this study, maps to a chromosomal region overlapping with previously reported QTLs for *S. minor* resistance identified through genome-wide association study (GWAS) in a lettuce diversity panel that included both PI 251246 and ‘Salinas’^13^. The QTL detected here appears to be located approximately 2.2 Mb away from *qLDR7.4* QTLs identified by GWAS. However, resistance QTLs in PI 251246 differ from those found in ‘Eruption’^19^, where resistance is neither associated with high lignin or hemicellulose content or strong stem^14^ nor early bolting^7,19^.

### Resistance independent of maturity

The intriguing results from the FA02 experiment, where QTLs for bolting and resistance did not overlap, identified 49 RILs that did not bolt by the end of the experiment (rating of 0). Notably, six of these RILs were among the most advanced bolting group in the spring season (rating of 4). Remarkably, these six lines also exhibited significantly lower disease incidence in the fall compared to other non-bolting RILs (21% vs. 28%, *p* = 0.023), even though none of the plants bolted by the end of the experiment. This suggests that factors beyond maturity may contribute to resistance in these lines. Further analysis revealed that these six lines had significantly higher total lignin (4.05 vs. 0.05) and syringyl lignin (2.52 vs. 0.22) content in SU17, when their values were adjusted for maturity. These findings further suggest that resistance in these RILs could be linked to higher lignin levels, particularly syringyl lignin, in stem cell walls, aligning with previous studies^14^. This opens the possibility of breeding for lignin-related resistance mechanisms, which could be independent of bolting time, offering a potential strategy to develop late bolting lettuce lines with high resistance to lettuce drop. Such an approach could be pivotal for breeding programs aiming to improve both disease resistance and delayed bolting.

### Lignin deposition, cross-linking, and other compounds potentially involved in resistance

Our current and previous findings^14^ indicate that elevated lignin content, particularly syringyl lignin, may enhance resistance to lettuce drop by contributing to stem strength. However, stronger and more resistant stems could also result from cross-linking between lignin and other cell wall components, such as cellulose and hemicellulose, without necessarily increasing the overall lignin or syringyl content^28^. Additionally, the location and timing of lignin deposition may play a crucial role, as seen in other crops such as *Brassica napus*, where earlier syringyl deposition in the vascular sclerenchymatic cortex tissue contributed to resistance against *S. sclerotiorum*^29^. The lignin content analyses conducted in this study would not detect these effects. Therefore, further investigation is needed into the mechanisms of lignin cross-linking, as well as the timing and location of lignin deposition. These mechanisms may explain the detection of a major QTL on LG7 from residuals to maturity, which was associated with increased resistance and stem strength but not with constituents of cell wall.

In addition to stem cell wall components identified here and in our prior studies^14^, other compounds have been linked to resistance in various plant species infected by *Sclerotinia* spp. For instance, resistance to *S. sclerotiorum* in soybean involves reprogramming the phenylpropanoid pathway and the accumulation of phenylpropanoid intermediates like cinnamic acid, ferulic acid, and caffeic acid^30^. A proteomic analysis of *Brassicaceae* also identified proteins involved in stem-physical-strength-mediated resistance to *S. sclerotiorum*, such as phenylalanine ammonia-lyase and cinnamyl alcohol dehydrogenase^31^.

### Bolting (maturity/stem elongation) and stem color QTLs

Four QTLs for plant maturity (bolting) detected in our field experiments were previously mapped in controlled environments that combined long (16 h) and short (8 h) day lengths with high (35 °C) and low (20 °C) temperatures^18^. The consistency of mapping bolting QTLs across natural and controlled environments underscores their stability. The major bolting QTL on LG2, previously linked to *Flowering Locus T* (*LsFT*)^32,33^, and the QTL on LG7 near *CONSTANS LIKE-9* (*LsCOL9*)^34^ and *PHYTOCHROME C* (*LsPhyC*)^35^ are central to lettuce flowering regulation^18^. Identical to a previous study^18^, alleles at QTLs for earlier bolting on LG6 and two on LG7 originated from PI 251246, while those on LG2 originated from cv. ‘Salinas’. For downstream validation, we plan on knocking out *LsFT*, *LsCOL9*, and *LsPhyC* genes to study their role in both bolting, stem mechanical strength and cell wall composition, and resistance to lettuce drop.

Stem color, exhibiting either green or red/purple coloration, showed a segregation pattern consistent with a 1:3 ratio (Chi-square *p* = 0.149). QTLs for stem color on LG5 and LG9 correspond to genes involved in anthocyanin biosynthesis. The QTL on LG5 overlaps with *RLL1*, a gene encoding a bHLH transcription factor, while the QTL on LG9 overlaps with the anthocyanin synthase (*ANS*) gene^36^. However, anthocyanins do not appear to play a significant role in lettuce drop resistance in this population, as resistance is more closely associated with SMS and CWC traits. This aligns with earlier studies that identified different genetic mechanisms in the resistance of cv. ‘Eruption’ and PI 251246 to *S. minor*^7,13,19,37^.

### Candidate genes for disease resistance

Several disease resistance proteins were identified in the regions of major QTLs on LG2 (Table 3), such as the disease resistance protein N, RGA3, and RPV1. This finding is not surprising, particularly because the distal part of LG2 is known to be a hotspot for a large number of R-genes^38,39^, as well as multiple QTLs linked to resistance against various pathogens^40,41^. It is possible that some of the resistance proteins found in this region may play a role in resistance to lettuce drop.

Of particular interest is the genomic region surrounding a major QTL on LG7, where QTLs for both disease resistance and stem strength were detected even after adjusting for plant maturity (Table 2; Fig. 3). One especially intriguing candidate gene in this region encodes *Callose synthase 5-like* protein, which is involved in synthesizing callose, a complex polysaccharide (specifically a β-1,3-glucan) composed of glucose molecules with occasional β-1,6-linked side branches. Callose serves several biological functions^42–44^, including contributing to mechanical strength in the cell wall and playing a critical role in the plant’s defense response to pathogen attacks. The accumulation of callose thickens the cell wall, forming a physical barrier against pathogen infection^45^. If callose is indeed involved in lettuce resistance to *S. minor*, it could help explain the close relationship between early bolting and disease resistance. A candidate gene for *Callose synthase* is located approximately within 2.5–3 Mb of three prominent candidate genes associated with plant maturity, bolting, or flowering. These genes include *Phytochrome C* (*LsPhyC*), which regulates flowering time and developmental transitions including bolting through light signaling pathways^46^; *Gibberellin 2-beta-dioxygenase 1*, a key enzyme in gibberellin catabolism that influences flowering and bolting^47^; and *CONSTANS LIKE-9* (*LsCOL9*), a regulator of flowering time^33^. Although the physical proximity of these genes to *Callose synthase* suggests possible co-expression of early bolting and resistance phenotypes, it also indicates the potential for these traits to be genetically separable. To explore this hypothesis further, we plan to conduct expression and functional studies on the candidate genes in the region of the major QTL on LG7.

The *Callose synthase 5-like* locus in cv. ‘Salinas’ (LOC128127382 on NCBI) is 872 base pairs (bp) long and comprises four exons. Alignment of this locus from ‘Salinas’ with available sequences (partial coverage of the coding regions) from PI 251246 (NCBI accessions SRX9023063, SRX9023064) revealed at least 11 SNPs (Supplementary Fig. 2). Of these, 7 SNPs are located within the coding sequence (CDS) across two exons. The putative protein sequence in cv. ‘Salinas’, derived by translating its CDS, is 106 amino acids long. At least four of the identified SNPs result in amino acid substitutions: Serine to Arginine, Glutamine to Arginine, Proline to Leucine, and Valine to Alanine (Supplementary Fig. 3), potentially affecting the protein’s function.

## Conclusions

Our findings highlight the central role of stem elongation in influencing resistance to lettuce drop within the studied population. Early bolting is associated with higher levels of pentose, xylose, and syringyl, along with lower levels of arabinose, stronger stems, and increased resistance. This underscores the influence of maturity on these traits, as indicated by path analysis. Critical QTL regions on LG2, LG6, and LG7 were found to colocate for traits related to maturity, SMS, and resistance, indicating that these loci are pivotal in controlling relationship between maturity, stem strength, and disease resistance in lettuce.

Further analyses using residuals to bolting revealed that some genetic effects on SMS and resistance may be independent of maturity, particularly at QTLs on LG7. This suggests a complex genetic architecture in which some regions affect stem strength and disease resistance without the influence of maturity. The combination of direct and independent genetic effects implies that, while early bolting is typically linked to resistance, it may be possible to select genetic variants that decouple these traits.

Several candidate genes were identified in the QTL region on LG7, with *Callose synthase* emerging as the most promising candidate gene involved in resistance to lettuce drop. The information about the *Callose synthase 5-like* locus, its sequence characteristics, and the SNPs between ‘Salinas’ and PI 251246 provides further support for its potential role. Our research indicates that, through careful selection of genotypes based on resistance traits such as SMS and CWC, breeding programs may develop late bolting lettuce cultivars with enhanced resistance to *S. minor*. Special attention must be given to increasing resistance-related compounds only in non-consumed tissues, such as the stem base, to avoid compromising the quality of lettuce for consumption.

## Electronic supplementary material

Below is the link to the electronic supplementary material.

Supplementary Material 1

Supplementary Material 2

## Data Availability

The datasets generated during and/or analyzed during the current study are available from the corresponding author on reasonable request.
