# Supplementary material for: Integrative path modeling and QTL mapping identify maturity, stem strength, and cell wall composition driving lettuce resistance to Sclerotinia minor
Source: Sci Rep. 2025 Jun 5;15:19824. doi: 10.1038/s41598-025-03775-1 (PMC12141734; doi:10.1038/s41598-025-03775-1)
Supplement: Supplementary file 1 — Supplementary Material 1 [file 41598_2025_3775_MOESM1_ESM.pdf]

Supplementary Figures 1 – 3.

Integrative path modeling and QTL mapping identify maturity, stem strength, and cell wall composition driving lettuce resistance to *Sclerotinia minor*

Ivan Simko[1,*], Bullo Erena Mamo[2,7], Shane L. Cantu[3], Hui Peng[4], Rebecca Grube Sideman[5], Ryan J. Hayes[6], Krishna V. Subbarao[2]

[1] United States Department of Agriculture, Agricultural Research Service, Sam Farr United States Crop Improvement and Protection Research Center, Salinas, CA 93905, USA

[2] Department of Plant Pathology, University of California, Davis, c/o Sam Farr United States Crop Improvement and Protection Research Center, Salinas, CA 93905, USA

[3] Department of Energy Great Lakes Bioenergy Research Center, Michigan State University, East Lansing, MI 48824, USA

[4] Everglades Research and Education Center – Horticultural Sciences Department, University of Florida, Belle Glade, FL 33430, USA

[5] Department of Agriculture, Nutrition and Food Systems, University of New Hampshire, Durham, NH 03824, USA

[6] United States Department of Agriculture, Agricultural Research Service, Forage Seed and Cereal Research Unit, Corvallis, OR 97321, USA

[7] Present address: Department of Microbiology and Plant Pathology, University of California, Riverside, CA 92521, USA

[*] Corresponding author, Ivan Simko, Ivan.Simko@usda.gov

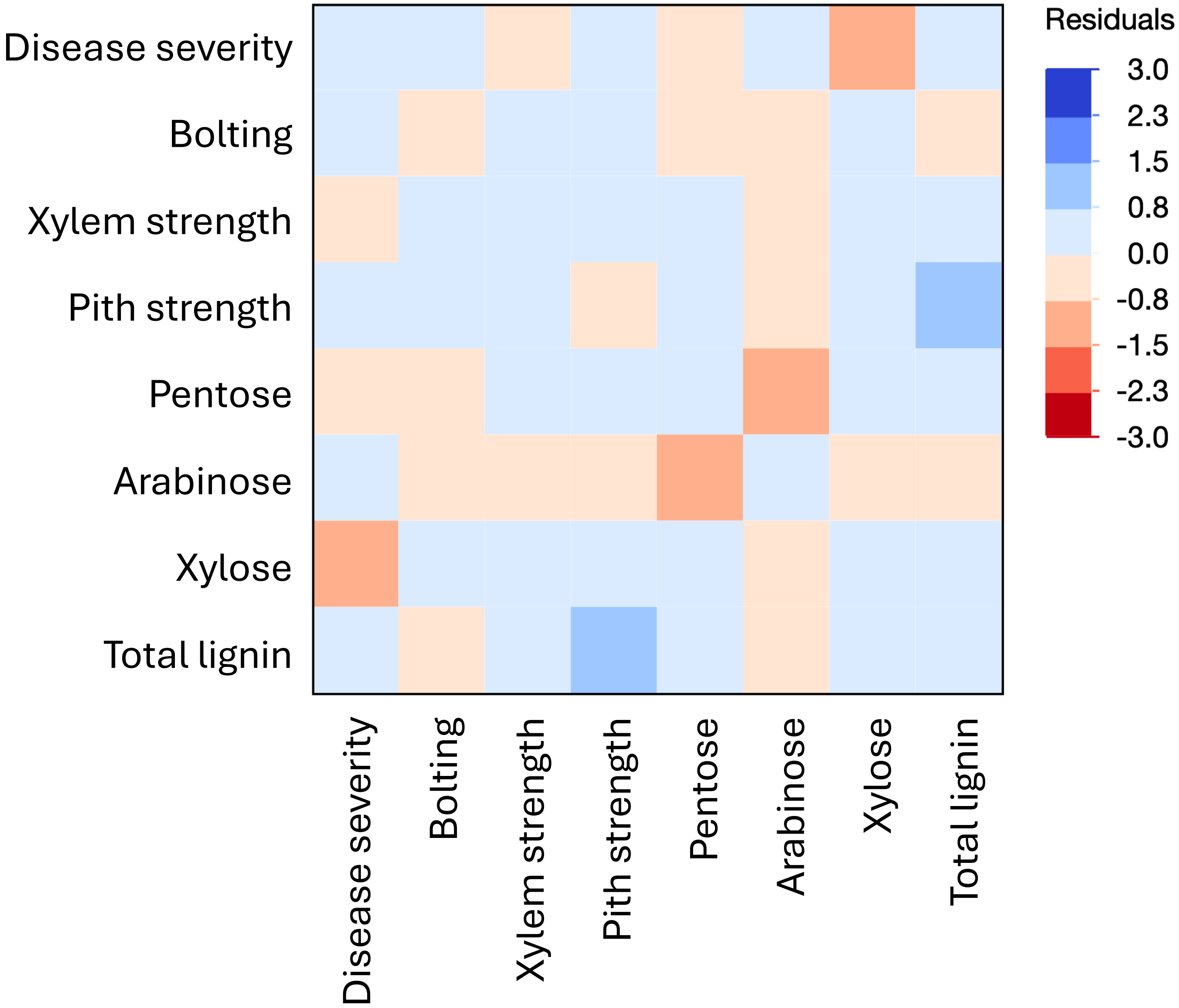

Supplementary Fig. 1. Normalized residuals heat map from path analysis.

The heat map displays the difference between the observed data, and the data that the model predicts.

```
Salinas           1 TCAGCCTGCT GCTTCTCTTT CTTCACTTGA TCACCGGATT CATCATCGTT    50
PI251246            ---------- ---------- ---------- ---------- ----------

Salinas          51 AGATTTCTGT ATCAAGTGTT TCTAACTGAG AAAAAGGGTT GAGTTCATAG   100
PI251246            ---------- ---------- ---------- ---------- ----------

Salinas         101 ATTCAATGAT TGGGGACTCC GAAGACTCGC ATACAAGATC CAAAAAGCAA   150
PI251246            ---------- ---------- ---------- ---------- ----------

Salinas         151 AAAAATGCAC ATTACATCTT GATGAACAGT TTTGTCAATT GTTGTTTGCC   200
PI251246            ---------- ---------- ---------- ---------- ----------

Salinas         201 GAAAGTGTAG TGGTGAAAGG CCAAATATGC GATTTTACCT AAAAGACTTC   250
PI251246            ---------- ---------- ---------- ---------- ----------

Salinas         251 CTGCATTACC CATGATTGAA ACATGTTAAT CACTCCAAGG TGGCTTATGT   300
PI251246            ---------- ---------- ---------- ---------G TGGCTTATGT

Salinas         301 TGAAGAGCGG GAAGAATCAG TTGATTTACT CACCCTTTTT AGTCGAAGCA   350
PI251246            TGAAGAGAGG GAAGAACCGG TTGA------ ---------- ----------

Salinas         351 GTCAACAGGT TTGACCAGGT AAGCTACCAA AACCAAAAAA TAAAGTCACA   400
PI251246            ---------- ---------- --------AA AACCAAACAA TAAACTCACA

Salinas         401 AACTTCACCA CTATAATTGT TATGTGGTGT GATTACAGGA GATATATCGC   450
PI251246            AACTTCAGCA CTATAATTGT TGTGTGGTGT GATTACAGGA GATATATCGG

Salinas         451 GTGAAGCTTC CAGGACCACC TAATATTGGA GAAGGGAAAC CTGAAAATCA   500
PI251246            GTGAAGCTAC CAGGACCACC TAATATTGGA GAAGGGAAAC TTGAAAATCA

Salinas         501 AAACCATGTC ATGTCTTTTG GATTATTATG TCTGTCTCTA ATCTGTACTT   550
PI251246            AAACCATGCC ATGTCTTTTG GATTATTATG TCTGTCTCTA ATCTGTACTT

Salinas         551 CACTTTGACA TCTTTTATTG AGTTACTTTC TTTCATGAAA TGTGAGCATT   600
PI251246            CACT------ ---------- ---------- ---------- ----------
```

```
Salinas          601 TACCGACATG CTATTTCCAG ATAAGGGGAG TAGGGGAGAA GCTGAGAAAG    650
PI251246             ---------- ---------- ---------- ---------- ----------

Salinas          651 CTGACAAGAA AGCTGATGAA AATGATGAAG GCCAACCACT TCCAGGAACT    700
PI251246             ---------- ---------- ---------- ---------- ----------

Salinas          701 GCTAGACATA GGGAATTTTA TCCGGAAGGT GTAGCTAAGG TCCTACAGGT    750
PI251246             ---------- ---------- ---------- ---------G TCCTACAGGT

Salinas          751 TAGTTTCATC CTTACTTCTT TTTTGATTGT TTTATATATA TATATATATA    800
PI251246             TAGTTTCATC CTTA------ ---------- ---------- ----------

Salinas          801 TATATATATA TATATATATA TATATATATA TATATATATA TATATATATA    850
PI251246             ---------- ---------- ---------- ---------- ----------

Salinas          851 TATATATATA TGAAAGAGAT GA    872
PI251246             ---------- ---------- --
```

Supplementary Fig. 2. Comparison of nucleotide sequences of the *Callose synthase 5-like* locus in cv. 'Salinas' and PI 251246.

Nucleotide sequence alignment of the *Callose synthase 5-like* locus from cv. 'Salinas' (LOC128127382 on NCBI) and PI 251246 (based on NCBI accessions SRX9023063, SRX9023064). Yellow highlighting indicates coding sequence (CDS). Red and underlined nucleotides denote differences between the two lines. Dashes indicate regions where data are not available in NCBI or where no significant match was found using BLAST.

```
Salinas            1 MLITPRWLML KSGKNQLIYS PFLVEAVNRF DQEIYRVKLP GPPNIGEGKP     50

PI251246             -----RWLML KRGKNRLI-- ---------- --EIYRVKLP GPPNIGEGKL


Salinas           51 ENQNHVMSFG LLYKGSRGEA EKADKKADEN DEGQPLPGTA RHREFYPEGV    100

PI251246             ENQNHAMSFG LLY------- ---------- ---------- ----------


Salinas          101 AKVLQR*    106

PI251246             --VLQ--
```

Supplementary Fig. 3. Comparison of predicted amino acid sequences of the *Callose synthase 5-like* protein in cv. ‘Salinas’ and PI 251246.

Predicted amino acid sequence alignment of the *Callose synthase 5-like* protein from cv. ‘Salinas’ (LOC128127382 on NCBI) and PI 251246 (based on NCBI accessions SRX9023063, SRX9023064). Red and underlined amino acids indicate differences between the two lines. Dashes represent regions where data are not available.
